# Global dispersal of *Burkholderia pseudomallei* and the evolving endemicity of melioidosis in the United States of America

**DOI:** 10.1371/journal.pntd.0014217

**Published:** 2026-04-24

**Authors:** Bart J. Currie, Mirjam Kaestli, Ella M. Meumann

**Affiliations:** 1 Global and Tropical Health Division, Menzies School of Health Research, Charles Darwin University, Darwin, Northern Territory, Australia; 2 Department of Infectious Diseases, Royal Darwin Hospital, Darwin, Northern Territory, Australia; 3 Microbiology Department, Territory Pathology, Darwin, Northern Territory, Australia; Institute of Continuing Medical Education of Ioannina, GREECE

## Abstract

Melioidosis is a clinical disease in humans and animals following infection with the soil and water bacterium *Burkholderia pseudomallei*. The global footprint of melioidosis has been rapidly expanding, but it remains unclear how much this represents unmasking of longstanding but previously unrecognised presence of *B. pseudomallei* and how much is from recent dispersal of *B. pseudomallei*. What is now clear is that the predicted establishment of *B. pseudomallei* in the southern United States has eventuated, with melioidosis endemic in Mississippi and likely to be endemic in Georgia and Texas. It is now time to move beyond concern of *B. pseudomallei* as a biothreat agent and to pivot towards addressing the gaps in public health responses to the enigmatic disease that it causes.

## Introduction

**Fig 1:**
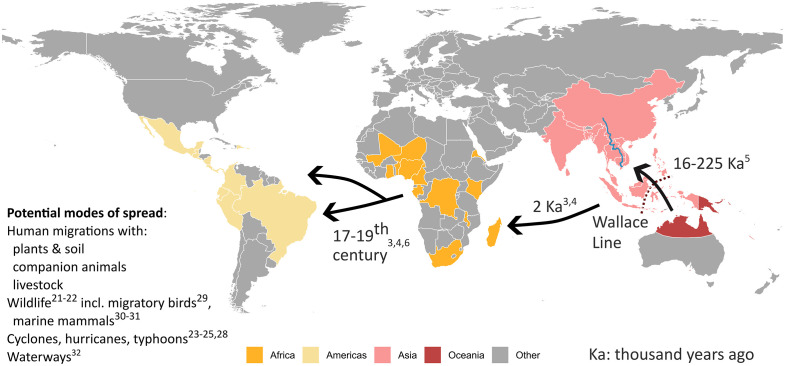
The global spread of *Burkholderia pseudomallei.* Basemap from Natural Earth (public domain) using the rnaturalearth R package. Data: https://www.naturalearthdata.com/downloads/10m-cultural-vectors/10m-admin-0-countries/, https://www.naturalearthdata.com/downloads/10m-cultural-vectors/10m-admin-1-states-provinces/, Terms: https://www.naturalearthdata.com/about/terms-of-use/.

Melioidosis is a clinical disease in humans and animals following infection with the soil and water bacterium *Burkholderia pseudomallei*. Global collaborations enabled identification of endemicity of melioidosis in 48 countries as of 2015 [1], with a further 12 countries confirmed as endemic by 2024 [2]. International collaborations have also facilitated phylogenetic studies of geolocated *B. pseudomallei* to track regional and intercontinental spread of melioidosis, with molecular clock analyses allowing estimated timelines for the global dissemination of this sapronotic bacterium [3,4]. Phylogenetic data support an Australian origin for *B. pseudomallei*, with spread to Southeast Asia estimated to have occurred during the last ice age when sea levels were much lower and land bridges joined Australia to Papua New Guinea and connected many of the current islands in the Malay Archipelago (Fig 1) [5]. Subsequent spread has been linked to population movements; from Asia to Africa estimated at two millennia ago and from Africa to the Americas in the 17th–19th centuries, potentially implicating the transatlantic slave trade [3,4,6].

### Melioidosis and *Burkholderia pseudomallei* in the United States of America

**Fig 2:**
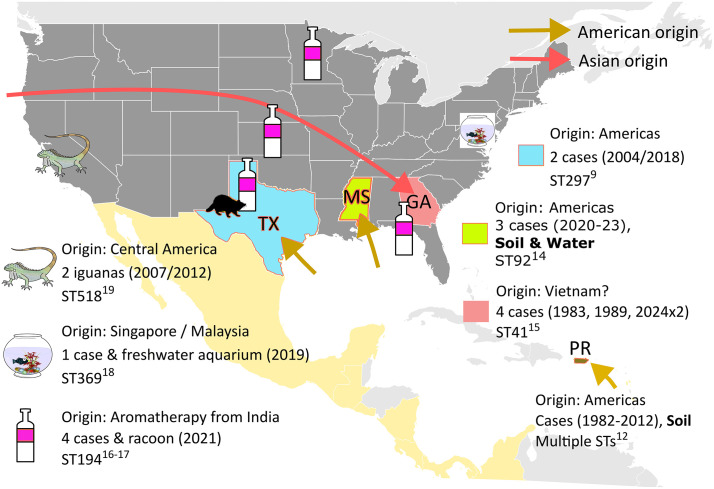
Melioidosis in the United States. Basemap from Natural Earth (public domain) using the rnaturalearth R package. Data: https://www.naturalearthdata.com/downloads/10m-cultural-vectors/10m-admin-0-countries/, https://www.naturalearthdata.com/downloads/10m-cultural-vectors/10m-admin-1-states-provinces/, Terms: https://www.naturalearthdata.com/about/terms-of-use/.

While melioidosis was historically known as an exotic tropical disease of Southeast Asia and northern Australia, the confirmed data from 2015 showed a far greater global distribution and modelling suggested an even greater potential distribution [1]. Notably, this study also included the predicted receptivity of the southern United States of America (U.S.), especially Gulf States, to environmental establishment of *B. pseudomallei* with subsequent endemic melioidosis. Around 12 cases of melioidosis in the continental U.S. are reported each year to the Centers for Disease Control and Prevention (CDC) [7,8]. While these melioidosis cases are primarily in people travelling or returning from known melioidosis-endemic regions, a small number of cases could not be linked to overseas travel or contaminated imported products. In one case of suspected locally acquired melioidosis in Texas from 2018, environmental sampling was conducted but was unable to confirm the presence of *B. pseudomallei* (Fig 2) [9,10]. Phylogenomic analysis of *B. pseudomallei* from the Texas case and two other historical cases from Texas and Arizona placed those 3 isolates in the “Americas” clade (Western Hemisphere), supporting introduction of *B. pseudomallei* to Texas from South or Central America or the Caribbean [9,10]. Concurrently, there has been increasing evidence of both unmasking and expansion of endemic melioidosis in South and Central America and the Caribbean, including Puerto Rico and the US Virgin Islands [6,11–13].

Endemicity of melioidosis in the continental U.S. was finally confirmed when 3 cases were reported from Mississippi between 2020 and 2023 (Fig 2) [14]. Genotyping of the clinical *B. pseudomallei* isolates showed that the patients were infected by an almost identical Western Hemisphere (Americas) *B. pseudomallei* strain to those cultured from soil and water samples from the local environment [14]. The very closely related genomes of the clinical and environmental isolates support a point-source introduction from South or Central America or the Caribbean, which may involve the rivers and their tributaries in that region of the Gulf. Possibilities include dispersal by flooding such as occurred after Hurricane Katrina and shipping-related activity such as bilge water release.

The most recent report of likely endemic melioidosis in the U.S. is from the state of Georgia, with bacterial isolates from 2 human cases in September 2024 following category 4 Hurricane Helene (Fig 2) [15]. Both patients had the same *B. pseudomallei* genotype, sequence type (ST) 41. Importantly, interrogation of the CDC multidecade surveillance archive of *B. pseudomallei* isolates identified 2 further historical ST41 cases in Georgia in 1983 and 1989. Both cases were fatal, and both were living in the same county as the 2 cases from 2024. Despite the cases spanning four decades, the genomes of the 4 Georgia *B. pseudomallei* ST41 isolates were all closely related, being separated by <20 single nucleotide polymorphisms (SNPs) [15]. ST41 is of Southeast Asian and not Americas origin and the closest ST41 *B. pseudomallei* isolates to the Georgia strains are from Vietnam [15].

There are military facilities in Georgia near where all 4 patients were likely infected and a reasonable hypothesis is that, unlike the presumptively more recent introduction of *B. pseudomallei* into Mississippi, *B. pseudomallei* may have entered Georgia decades ago with returning troops and equipment following the Vietnam War and become established in the receptive local environment (Fig 2) [15]. The presence of *B. pseudomallei* in the local environment was presumably unmasked by the severe weather event of 2024.

Melioidosis should therefore now be considered endemic in Mississippi and is likely endemic in Georgia and Texas, although *B. pseudomallei* is yet to be cultured from the environment in the latter two states [9,10,14,15].

Adding further complexity to melioidosis epidemiology are specific examples of unexpected melioidosis cases in the continental U.S. that have been genomically linked to *B. pseudomallei* imported from overseas. Four cases from four separate states were traced to *B. pseudomallei* contaminated aromatherapy spray imported from India (Fig 2) [16]. Inhalation was the likely route of infection which in part explains the high mortality and morbidity: 2 fatalities including a child and another child surviving with severe residual neurological disability. A pet racoon also succumbed; the animal had broken a bottle of the implicated aromatherapy spray and subsequently died from likely neurological melioidosis (Fig 2) [17]. A single case of melioidosis from Maryland was traced to a freshwater home aquarium that had contained imported tropical fish, with *B. pseudomallei* genotyping supporting Southeast Asian origin for the fish and contaminated water (Fig 2) [18]. Various imported animals have been diagnosed with melioidosis, including primates from Southeast Asia and pet iguanas from Central America (Fig 2) [19]. In none of these other examples of imported melioidosis was there evidence of environmental persistence of *B. pseudomallei*, although the aromatherapy-related racoon death necessitated a public health response to excavate the buried carcase and decontaminate the surrounding soil [17].

### How is global dissemination of *Burkholderia pseudomallei* occurring?

What remains unclear is how much of the global footprint of melioidosis represents unmasking of previously unrecognised presence of environmental *B. pseudomallei* that was established long in the past and how much is from recent intercontinental and regional dissemination of *B. pseudomallei* [20]. While the Americas represent currently the most dynamic region globally for dispersal, the timelines and modes of dissemination of *B. pseudomallei* in the Americas remain unclear [2,3].

Historical intercontinental spread of melioidosis has coincided with movements of people with their animals and plants [3,4] and importation of infected animals has been documented (Fig 1) [19,21,22]. Aerosol spread linked to severe weather events such as hurricanes, cyclones, and typhoons has also been hypothesised [23–25]. Limited air sampling studies have confirmed that *B. pseudomallei* can be recovered during windy and rainy conditions [26,27], however *B. pseudomallei* is sensitive to ultraviolet radiation [28]. Birds can be colonised with *B. pseudomallei*, raising the surprising but plausible potential for long distance dispersal by migratory birds using flyways such as the great East Asian–Australasian Flyway [29]. Similarly, marine mammals are susceptible to melioidosis and have the potential to carry *B. pseudomallei* over long ocean distances between continents (Fig 1) [30,31]. The global phylogeographical separation of isolates by region suggests that *B. pseudomallei* is not readily carried long distances by these modes of dispersal. There is, however, increasing evidence for the role of rivers such as the Mekong River in Southeast Asia in dispersing *B. pseudomallei* within the region (Fig 1) [32].

**Fig 3:**
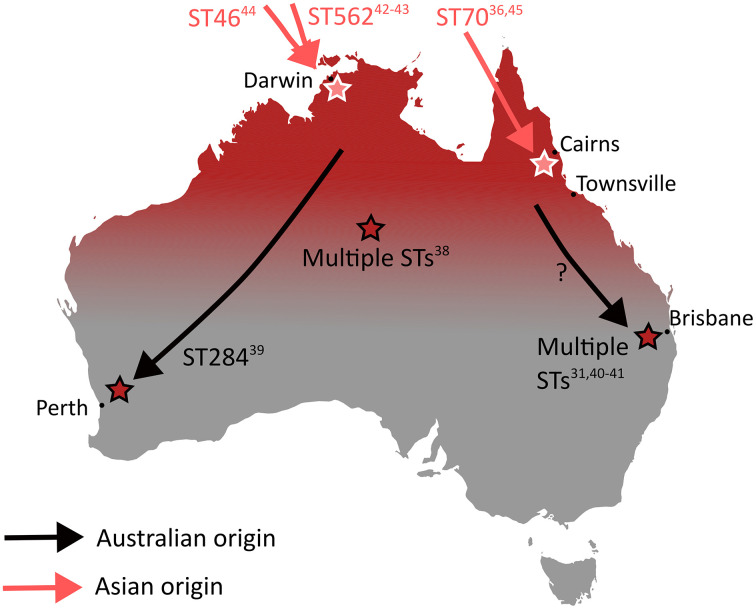
Melioidosis in Australia. Basemap from Natural Earth (public domain) using the rnaturalearth R package. Data: https://www.naturalearthdata.com/downloads/10m-cultural-vectors/10m-admin-0-countries/, https://www.naturalearthdata.com/downloads/10m-cultural-vectors/10m-admin-1-states-provinces/, Terms: https://www.naturalearthdata.com/about/terms-of-use/.

While the global phylogeographical analyses provide robust support for the intercontinental spread of *B. pseudomallei* from Australia to Asia to Africa to the Americas, analyses of *B. pseudomallei* from within Australia show complexities at a within-continent level, reflecting the ancient origins of *B. pseudomallei* and the subsequent massive diversity seen (Figs 1 and 3) [5,33–37]. Separated from the melioidosis-endemic regions of tropical northern Australia are three sub-tropical foci of endemic but sporadic melioidosis documented in Australia, with cases only occurring in some years and always after heavy rainfall (Fig 3) [31,38–41]. These demonstrate the wide diversity of climatic and environmental conditions under which *B. pseudomallei* can persist once introduced into a region.

Confounding the Australian *B. pseudomallei* phylogenomics and a harbinger of the future global footprint of melioidosis on a warming planet where people, animals, and products are increasingly on the move, is the recent documentation of 3 separate introductions of *B. pseudomallei* from Asia to northern Australia; ST 562, ST 46, and ST 70 (Fig 3) [36,42–45]. Each is likely to reflect a point-source introduction from Asia, but the specific origin locations of these 3 introductions of *B. pseudomallei,* the mode of introduction for each and the introduction locations in northern Australia all remain unknown. All 3 are now being found amongst new patients with melioidosis, confirming the establishment of these newly endemic “Asian” STs amongst the long established Australian STs.

### Implications for the U.S. and globally

Currently, *B. pseudomallei* is listed in the U.S. as a Tier 1 select agent, subject to strict reporting and handling as a potential biothreat pathogen [8,46]. This reflects the triad of melioidosis being able to cause rapidly fatal disseminated sepsis, the capacity for *B. pseudomallei* to be aerosolised and the bacterium’s intrinsic resistance to many antimicrobials.

The concern of *B. pseudomallei* weaponization has prompted funding in the U.S. and other countries that has led to major advances in diagnosis and therapy of melioidosis, as well as advances in understanding the complex global epidemiology. With melioidosis now confirmed as endemic in the U.S., it is time to pivot to addressing the gaps in public health aspects of this enigmatic disease [47]. Continuing the current requirement to handle *B. pseudomallei* in a physical containment level 3 laboratory is likely to impact timely and accurate melioidosis diagnosis, with delayed or incorrect initial species identification recognised as a problem in the U.S. as occurs elsewhere [14,17,46,48].

The CDC has been providing critical expert advice for U.S. laboratories and clinicians when suspected or confirmed cases are identified [8,46] and has also supported the environmental sampling and epidemiological investigations around the Texas, Mississippi, and Georgia cases [9,10,14,15,17].

With the recent expansion of melioidosis in the Americas and confirmation of endemicity in at least 1 U.S. Gulf State, there are public health, laboratory, and clinical management challenges to address in the U.S. [49]. These have already required substantial resourcing in the historically endemic regions of Asia and northern Australia [1,2]. The first priority is public and health staff awareness of melioidosis and of the clinical risk factors that make individuals susceptible, and the occupational and recreational activities that can result in infection. Second is laboratory diagnosis, laboratory safety, and clinician management of patients, with complex and prolonged antimicrobial regimens required for therapy of melioidosis [2]. This remains a global challenge, with the unmasking of melioidosis in Africa demonstrating the critical importance of improving availability of and access to microbiology laboratories and especially blood culture capacity [50]. This is highlighted by the recent finding in Mali of previously undocumented high rates of bacteremic melioidosis in children [51]. Melioidosis has a wide spectrum of clinical presentations that justify it being recognised as one of the great mimickers amongst infectious diseases, alongside tuberculosis which it is often initially mistaken for. Finally, it is critical for the U.S. and global health communities to maintain and support the strong partnerships addressing all the facets of melioidosis, led by the International Melioidosis Network (https://www.melioidosis.info/). This will only become more important as *B. pseudomallei* spreads to or is unmasked in new locations, with the predicted inexorable rise in case numbers of melioidosis driven by the worldwide increases in people living with diabetes and by severe weather associated with climate change.

**Key learning points**

- *Burkholderia pseudomallei* is being unmasked in previously unrecognised endemic locations, such as in Africa, but it is also spreading globally.
- Melioidosis has spread to the USA and is now endemic in Mississippi.
- Melioidosis may also be endemic in Georgia and Texas.
- Public health messaging, laboratory awareness, and clinician therapeutic guidance will decrease morbidity and mortality.
- Systematic clinical surveillance and targeted environmental sampling will help define the local epidemiology and track future expansion of endemicity in the USA and elsewhere.

**Top five papers**

1. Limmathurotsakul D, Golding N, Dance DAB, et al. Predicted global distribution of *Burkholderia pseudomallei* and burden of melioidosis. Nat Microbiol. 2016; 1:15008.
2. Meumann EM, Limmathurotsakul D, Dunachie SJ, Wiersinga WJ, Currie BJ. *Burkholderia pseudomallei* and melioidosis. Nat Rev Microbiol. 2024; 22(3):155–169.
3. Chewapreecha C, Holden MT, Vehkala M, et al. Global and regional dissemination and evolution of *Burkholderia pseudomallei*. Nat Microbiol. 2017; 2:16263.
4. Petras JK, Elrod MG, Ty MC, et al. Locally acquired melioidosis linked to environment - Mississippi, 2020–2023. N Engl J Med. 2023; 389(25):2355–2362.
5. Brennan S, Thompson JM, Gulvik CA, et al. Related melioidosis cases with unknown exposure source, Georgia, USA, 1983–2024. Emerg Infect Dis. 2025; 31(9):1802–1806.
